# Frailty Screening and Case-Finding for Complex Chronic Conditions in Older Adults in Primary Care

**DOI:** 10.3390/geriatrics3030039

**Published:** 2018-07-07

**Authors:** Linda Lee, Tejal Patel, Loretta M. Hillier, Jason Locklin, James Milligan, John Pefanis, Andrew Costa, Joseph Lee, Karen Slonim, Lora Giangregorio, Susan Hunter, Heather Keller, Veronique Boscart

**Affiliations:** 1 Centre for Family Medicine Family Health Team, Kitchener, ON N2G 1C5, Canada; t5patel@uwaterloo.ca (T.P.); jason.locklin@family-medicine.ca (J.L.); james.milligan@medportal.ca (J.M.); john.pefanis@gmail.com (J.P.); joe.lee@family-medicine.ca (J.L.); karen.slonim@gmail.com (K.S.); 2 Department of Family Medicine, McMaster University, Hamilton, ON L8P 1H6, Canada; 3 Schlegel—University of Waterloo Research Institute for Aging, University of Waterloo, Waterloo, ON N2J 0E2, Canada; lora.giangregorio@uwaterloo.ca (L.G.); hkeller@uwaterloo.ca (H.K.); vboscart@conestogac.on.ca (V.B.); 4 School of Pharmacy, University of Waterloo, Waterloo, ON N2G 1C5, Canada; 5 Geriatric Education and Research in Aging Sciences (GERAS), Hamilton Health Sciences, Hamilton, ON L8M 1W9, Canada; lmhillier@rogers.com; 6 Departments of Clinical Epidemiology & Biostatistics, and Medicine, McMaster University, Hamilton, ON L8S 4K1, Canada; acosta@mcmaster.ca; 7 Department of Kinesiology, University of Waterloo, Waterloo, ON N2L 3G1, Canada; 8 School of Physical Therapy at Western University, London, ON N6G 1H1, Canada; smuir4@uwo.ca; 9 Schlegel Centre for Advancing Seniors Care, Conestoga College, Kitchener, ON N2G 4M4, Canada

**Keywords:** frailty, screening, case-finding, comorbid conditions, primary care

## Abstract

With the aging population, escalating demand for seniors’ care and limited specialist resources, new care delivery models are needed to improve capacity for primary health care for older adults. This paper describes the “C5-75” (Case-finding for Complex Chronic Conditions in Seniors 75+) program, an innovative care model aimed at identifying frailty and commonly associated geriatric conditions among older adults within a Canadian family practice setting and targeting interventions for identified conditions using a feasible, systematic, evidence-informed multi-disciplinary approach. We screen annually for frailty using gait speed and handgrip strength, screen for previously undiagnosed comorbid conditions, and offer frail older adults multi-faceted interventions that identify and address unrecognized medical and psychosocial needs. To date, we have assessed 965 older adults through this program; 14% were identified as frail based on gait speed alone, and 5% identified as frail based on gait speed with grip strength. The C5-75 program aims to re-conceptualize care from reactive interventions post-diagnosis for single disease states to a more proactive approach aimed at identifying older adults who are at highest risk of poor health outcomes, case-finding for unrecognized co-existing conditions, and targeting interventions to maintain health and well-being and potentially reduce vulnerability and health destabilization.

## 1. Introduction

Canada’s population is aging, with the number of older adults expected to double over the next two decades [1]. Yet Canada faces a critical shortage of geriatricians [2,3] and wait times to access specialist care can be lengthy–commonly 6 to 12 months [4]. It is increasingly recognized that primary care has an important role to play in the management of chronic diseases. Primary care, in comparison with specialist care, can increase equitable access to care, reduce care costs with early community-based interventions that can prevent crises that result in hospitalization and increase access to more appropriate services, resulting in improved health outcomes [5]. In managing complex chronic conditions of older adults, primary care is particularly well-positioned to provide person-centred care [6]. Recently, the importance of a person-centered care approach has been emphasized to meet the aims of improving healthcare safety, quality and coordination, as well as quality of life for older adults [7], and, in particular, to meet the needs of frail older adults [8,9].In addition, there is growing recognition of the clinical significance of frailty in primary care [10]. 

Frailty has been defined as a state of increased vulnerability from age-associated decline in reserve and function resulting in reduced ability to cope with everyday or acute stressors [11,12]. Frailty places older adults at greater risk for adverse outcomes [13]. Frailty has been associated with increased risk of recurrent falls, fractures and disability [14] as well as increased health service utilization [15,16] and mortality [13,17,18]. Given the dynamic, multidimensional aspects of frailty and disability involving physiologic, psychological, social and environmental factors [10], primary care practitioners are in a unique position to consider the effect of multimorbidity in the context of the person’s individual circumstances and to tailor treatment recommendations to realistically attainable healthcare goals [6]. However, frailty is easily overlooked by primary care practitioners because its manifestations can be subtle and slowly progressive, and thus dismissed as normal aging. Yet frailty is common, particularly in elderly persons with complex chronic conditions. Some degree of frailty may be present in more than half of older persons with heart failure (HF) [15,19] or chronic obstructive pulmonary disease (COPD) [20]. Frailty is present in an estimated 32% of persons in the community with Alzheimer’s disease, and cognitive impairment is present in up to 22% to 40% of persons who are identified as frail [21,22]. Older persons with urinary incontinence are 6.5 times more likely to be frail compared with those who are continent [23]. Frailty is a significant predictor of future falls in community-dwelling older adults [14,21].

Further, frailty is emerging as a predictor of adverse outcomes in older adults [15,16,24,25]. It can be conceptualized as the convergence of geriatric and medical conditions, which along with other factors, such as socioeconomic circumstances can lead to the domino effect of worsening health, or health destabilization [26]. For primary care practitioners, recognition of frailty might help in the appropriate counseling of patients and family members when discussing risks of medical interventions. Identification of frailty can ensure treatment decisions consider the potential for worsened outcomes in the context of frailty, resulting in better-informed decision-making that is consistent with the individuals’ wishes and values [26]. As well, by recognizing coexisting conditions associated with frailty, primary care physicians can optimize management, potentially avoiding health destabilization and associated costly hospitalizations and emergency room (ER) visits. 

The escalating demand for seniors’ care requires the development of complementary care delivery models that optimize efficiency of use of limited available specialist resources and build capacity to meet the health care needs of older adults. Identification and management of frailty and associated complex chronic conditions of older adults must be integrated and sustained within primary care, yet it is well-recognized that these conditions are amongst the most difficult for primary care physicians to manage [27,28,29,30]. Moreover, given that frailty is a multidimensional concept with dynamic interrelated contributing factors [10,31,32], it is recognized that interventions must be multi-disciplinary, multi-faceted and holistic to meet the needs of frail older adults. Interventions that change the system of care may be most effective [33]. To our knowledge, there has been no standardized systematic approach to both identification and management of frailty and its associated complex chronic conditions within Canadian family practice.

To meet this need, the Centre for Family Medicine (CFFM) Family Health Team (FHT), located in Kitchener, Ontario, Canada, developed a systematic, multi-faceted approach to: (a) identify older adults who are frail and potentially at highest risk of poor health outcomes; and (b) offer them interventions that might reduce risk of health destabilization and avert medical crises that result in hospital use. In Ontario, Canada, FHTs consist of groups of family physicians and multidisciplinary health care providers, such as nurses, social workers, pharmacists, and occupational therapists, working together to provide a patient-centered approach to primary care [34]. Referred to as “C5-75” (CFFM Case-finding for Complex Chronic Conditions in Seniors 75+), this pro-active approach provides coordinated care that is integrated into the system of primary care and involves the spectrum of necessary health care disciplines. Patients 75 years of age and older were targeted for this program primarily due to feasibility based on the numbers of frail persons estimated in the family practice and due to resource limitations (funding and human resources). However, this care model could easily be expanded to include a younger target group, particularly as frailty can occur in younger adults [11]. The C5-75 program was designed to provide care rooted in primary care practice. This is particularly important because this is where most health care for older adults is delivered and ensuring integration into the routine course of primary care practice increases likelihood of sustainability. Moreover, in Canada, there is a shortage of specialists [2] that is predicted to worsen over time as about a third of the existing 350 geriatricians will reach retirement age within the next 10 years [3]. C5-75′s pro-active orientation to care recognizes that those complex conditions that are the highest drivers of health system resource use in older adults are also some of the most difficult to identify early on because there is no simple diagnostic test (unlike for diabetes or hypertension) and they are complex and take time and skill to diagnose early on. Thus, we believed the intervention needed to be systematic, implemented into the course of regular care using allied health professionals. We also did not want an intervention that was parallel to or separate from primary care, as that would serve to fragment care and likely would not be sustainable [33]. The primary objectives of the C5-75 program are summarized in Table 1.

This program has been successfully implemented in the CFFM FHT since 2012 and, in 2013, was named an Ontario Ministry of Health and Long Term Care Demonstration Project to Improve the Care of Medically Complex Patients. The CFFM FHT is an established academic Family Health Team and is comprised of 16 urban and 3 rural family physician practices and currently serves 28,309 patients, of whom 1432 are aged 75 or older.

In this paper, we describe the innovative C5-75 program of care which uses a systematic, evidence-informed multi-disciplinary approach aimed at identifying frailty and commonly associated geriatric conditions among older adults and targeting interventions accordingly. This program aims to build capacity within primary care to better identify and manage complex chronic conditions in frail seniors. In this paper, we describe how we developed the C5-75 program, the two screening levels (protocol and procedures), interventions used to manage identified conditions, and present the case-finding results of our program.

### The C5-75 Case-Finding Program

A first step in the development of the C5-75 program was to establish a feasible, objective, valid means to screen for frailty in the context of a busy primary care setting. Although multiple frailty scales have been developed to date, systematic reviews have not conclusively identified a preferred instrument for measuring frailty [35]. Our recent systematic review confirmed the lack of psychometrically sound and clinically useful frailty markers that have been validated in ambulatory care settings [36]. There has been general lack of consensus as to how best to screen for, assess, and diagnose frailty in primary care practice [37,38]. The Fried frailty phenotype measure has been extensively tested for its validity [35,39,40] and is widely used in frailty research [35,41,42,43]; yet care processes in typical primary care practice are not conducive to the administration of measures of frailty that are impracticable or time-intensive. Rather than composite measures such as the Fried criteria, the feasibility of assessing frailty in primary care might be facilitated by the use of single-trait measures. There has been concern, however, that single-trait measures of frailty such as gait speed alone may have moderate-to-low predictive value; that is, they may produce too many false positives [43,44]. We demonstrated that while single-trait measures of gait speed or hand grip strength alone were sensitive and specific proxies for the Fried frailty phenotype, use of gait speed with grip strength was accurate, precise, specific, and more sensitive than other possible combinations [44]. Measuring grip strength along with gait speed can increase screening precision without adding significant time, training or costs [45,46]. We concluded that use of the dual measure of gait speed and hand-grip strength could be used as a proxy for Fried frailty phenotype and could represent a practical and efficient means of screening for frailty within primary care. Thus, the dual trait measure of gait speed with hand-grip strength has been used as the screen for frailty in our C5-75 program. 

The C5-75 program is based on the chronic care model [47], a framework that has been widely adopted by healthcare organizations to improve the management of various chronic diseases in ambulatory care. The framework promotes six interrelated elements: (a) multidisciplinary care, (b) patient self-management, (c) coordinated care, (d) delivery system redesign, (e) clinical information systems, and (f) evidence-based care. By bringing together the patients’ family physician, interprofessional healthcare providers, specialist physicians (if necessary) and community resources, healthcare providers are integrated within the primary care practice to meet patient and caregiver needs. Consistent with an ideal chronic disease management approach, most chronic conditions should be managed with low-intensity interventions; only a small proportion of patients will need mid-intensity and high-intensity interventions [48]. Given the realities of busy primary care practice, with hectic workflow and time and resource limitations, comprehensive assessments could only happen at a separate dedicated visit. Thus, we developed a two-level screening system in which those who screen positive for frailty in the first level would then be offered a more comprehensive assessment at a separate second level visit. In addition, those at high risk of falls and those who have a diagnosis of HF are also scheduled for Level 2 screening.

We selected screening tools and interventions implemented in the C5-75 program and made revisions to screening processes and interventions, based on current research evidence, guideline-informed best practices, and clinical expert opinion from family physicians and specialists in geriatric medicine, respirology, cardiology, internal medicine and urology and academic experts in kinesiology, nursing, physiotherapy, nutrition, social work, geriatric pharmacology and gerontology. Regularly scheduled interdisciplinary meetings, usually held on a quarterly basis, were open to the researchers and clinicians that formed our project team. During these meetings, we reviewed screening results to date, discussed challenges and potential solutions, and made decisions about changes to screening tools used and where in the screening process they would be introduced (Level 1 or Level 2). In addition, several expert opinion meetings were held as needed to determine best practices related to specific conditions. During the course of data collection, meetings were again held with these clinical specialists to review the data and discuss the need for further changes to the screening tools and algorithms as well as changes to practice. A similar process was used to incorporate fracture risk assessment into the program. All screening tools and recommendations stemming from these expert consultations were reviewed with the larger project group members prior to implementation. The program has evolved through a continuous iterative process of balancing potential benefits of screening and interventions offered with acceptability and feasibility given limitations of time and human resources in a busy clinical practice setting. As a result, some screens that were trialed in the first iterations of the program are no longer implemented. An example of this process is illustrated with our HF screening, described in Figure 1. This practice improvement process is consistent with the knowledge-to-action (KTA) process to facilitate the development and application of research evidence into clinical practice [49].

## 2. Methods

### 2.1. Screening Protocol and Procedures

Since 2012, all persons aged 75 or older with medical appointments at the [Blinded] FHT were offered annual assessments through the C5-75 program during regular office visits. Both screening levels are completed by a registered nurse but can be completed by trained allied health care professionals. Level 1 screening (Table 2) is conducted annually for all patients aged 75 years and older in the office immediately preceding scheduled appointments with their family physician; it is deferred if the patient is acutely unwell, if they decline to be assessed, or if practice flow does not allow adequate time for the assessment. Level 1 involves screening for low physical activity, frailty, medical history or symptoms suggestive of HF or COPD, and falls. Frailty screening involves measurement of gait speed [55], calculated as the number of seconds to walk four meters at a usual pace, with the fastest time of two trials being recorded, and hand grip strength [56], which is measured as the higher score of two 3 s trials (with each hand) using a handheld dynamometer (Jaymar Hydraulic Dynamometer, Model #281-12-0600, J.A. Preston Corp, Clifton, NJ). Potential frailty is defined as four-meter gait speed of greater than six seconds [55]. Hand grip weakness is defined as a score within the lowest 20% of the population, and stratified by gender [44,57]. All persons with known HF are referred directly for a Level 2 screening and to a pharmacist for medication review as it was felt all persons with HF might benefit from a comprehensive assessment and assurance that medications are optimized. If persons are identified with Medical Research Council (MRC) dyspnea scale score ≥ 2, then the patient’s family physician is alerted that this might represent poorly controlled cardiorespiratory conditions and reassessment of the patient is recommended. All patients with a smoking history of at least 20-pack years are asked about respiratory symptoms based on Canadian Thoracic Society (CTS) Guidelines [58] for COPD screening, consisting of five questions; a positive response to at least one of these questions was considered a positive screen. Self-reported physical activity was assessed by asking patients to select from one of three descriptions that best matched their activity level [59] (Table 1). Screening for falls involves asking patients about their recent history of falls, near falls, and falls requiring medical attention [60]. Patients identified as frail patients are scheduled for Level 2 multidisciplinary screening at a separate office visit based on gait speed at Level 1, as well as those who have a history of falls (two or more in six months, or any falls requiring medical attention in six months) and those with known heart failure.

Level 2 screening is conducted by nursing, pharmacy, and if applicable, social work, using standardized measures known to have good psychometric properties. Level 2 (Table 3) consists of screening of the frail senior for nutrition, fracture risk [61], urinary incontinence [62,63,64], depression [65], anxiety [66], social isolation [67], caregiver burden if applicable [68], cognitive impairment [69], and falls risk [60]. In addition, at Level 2 all persons also receive a full medication review to identify medication-related problems, polypharmacy, and high-risk medication use [70]. In addition, the Assessment Urgency Algorithm (AUA) is administrated. The AUA is a standardized and validated algorithm that quickly and easily identifies the level of risk faced by older adults by classifying them into one of six risk levels with higher scores reflecting the need for more immediate intervention [71].

Level 1 takes approximately 7 min to complete [44] and Level 2 is estimated to take approximately 30 min to complete. Level 2 screening requires more time because it involves implementation of screening for many conditions associated with frailty as well as management interventions. Once completed, screening results are documented in a patient’s electronic medical record (EMR) with a customized form created for this purpose. Screening results are also maintained in a research-specific database that includes patient age, gender, and previous diagnosis of chronic conditions for the purpose of studying specific facets of frailty and chronic diseases in older adults. Approval for the maintenance of this database was obtained from the Hamilton Integrated Research Ethics Board, McMaster University (#13-602C). De-identified screening results from all Levels 1 and 2 screening sessions were collected and processed with R to produce summary statistics describing the results of the screening program to date [72].

### 2.2. Management Interventions

When screening reveals potential frailty or comorbid conditions that require management, we implement interventions to reduce vulnerability and prevent medical crises (Table 4). These interventions are based on current research evidence, best practice guidelines, and local clinical expert opinion from family physicians and from specialists in geriatric medicine, respirology, cardiology, internal medicine, and urology. The C5-75 program provides care aligned with essential elements of person-centred care [7] in that it is, for example, multidisciplinary team-based, facilitates an informed discussion with the family physician and patient about goals of care in the context of frailty, provides continual integrated information sharing with their primary care physician, and supports capacity building for family physicians and health education for patients. Moreover, identification of frailty can bring greater awareness to primary care physicians of the need for fulsome discussions with patients and families about the risks of proposed medical interventions in the context of frailty, carefully considering individual values, goals and preferences [26]. When new conditions have been identified, the patient’s family physician is notified through messaging via EMR as well as documentation in the patient’s EMR and the patient is referred to the appropriate care provider for further assessment, as needed, and management (Table 4). Where applicable, management suggestions are provided to the family physician. For example, if a patient is identified to be at high risk of fracture, a summary of the 2010 fracture prevention guidelines is sent to the family physician [61].

When potentially inappropriately prescribed medications or medication non-adherence have been identified, the family physician will be notified and team pharmacists will recommend medication changes, suggest safer alternatives for high-risk medications or recommend deprescribing. In other cases, patient education and information is provided. For example, for those identified with low levels of physical activity, we provide information on available local programs for exercise, balance and strength training. For those identified with urinary stress or urgency incontinence, we recommend pelvic physiotherapy. Patients identified with cognitive impairment are referred, as appropriate, to the FHT’s Primary Care Collaborative Memory Clinic for more intensive assessment and management of memory-related concerns [73,74]. Those identified at high risk of falls are referred to the Mobility Clinic for further management of gait or balance concerns resulting in falls [75]. When the Assessment Urgency Algorithm score indicates they are at highest risk of adverse outcome, a recommendation is made to the family physician for consideration of referral to a geriatric medicine specialist. For those screening positive for possible COPD based on smoking history and Canadian Thoracic Society screening recommendations, we refer patients to a certified Respiratory Therapist for spirometry. We refer patients identified with nutritional concerns to the FHT dietitian. External to the CFFM FHT, partnerships have been fostered with community agencies in providing supervised exercise programs and outreach workers to assist with engagement in community programs. 

## 3. Results

### Case-Finding Outcomes

A total of 1,461 Level 1 assessments were conducted between April 2013 and December 2016; 965 of these were unique patients (representing approximately 67% of individuals aged 75+ eligible patients in this practice setting at that time) and 496 were repeat annual assessments. Table 5 presents the characteristics of this patient population. Following identification from Level 1, 582 unique patients completed the Level 2 screening (Table 6); 640 screens were performed in total when repeat annual screens are included. Across the Level 1 screening components, a liberal selection of criteria have been used to trigger Level 2 comprehensive screening during iterative program development in order to provide sufficient opportunity for evaluation and calibration. All of those deemed frail based on gait speed, as well as, eventually, all patients identified with HF, were triaged for Level 2 screening. Conservatively, 178 Level 2 screens were necessary based on slow gait speed as a proxy indicator of frailty risk, which would represent a triage rate of 12%. Our next steps are to evaluate the sensitivity and specificity of the C5-75 screening components for identifying frailty and other chronic conditions and the effect of the program on potentially averting health destabilization and healthcare utilization.

## 4. Discussion

This paper describes a unique primary care approach to screening for and managing frailty and associated conditions in older adults. The C5-75 program moves away from single disease management after late diagnosis and towards a multi-faceted proactive approach to chronic disease management for older adults, using frailty to identify those at highest risk of poorest outcomes and integrating targeted multi-faceted care into the person’s system of primary health care. 

Frailty is common in older persons, and most frail persons have at least one chronic condition [83], with a higher number of chronic conditions associated with increased frailty risk. Although the prevalence of frailty varies greatly depending on the method of measurement [18,84], overall it is estimated to affect 10.7% of community-dwelling persons aged 65 and older. Prevalence of frailty increases with age, affecting an estimated 15.7% of those aged 80 to 84, and 26.1% of those aged 85 and older [85]. Consistent with this, our sample of 965 older adults over 75 years of age found that 7–14% of patients were potentially frail, depending on the method of screening (i.e., gait speed alone or combined with grip strength). As recent studies suggest a higher prevalence of frailty in persons with certain chronic conditions [15,20,21,22], identification of these conditions, as in the two C5-75 Program screening levels, provides an opportunity for managing these conditions. 

The management of complex chronic conditions in older adults is challenging for primary care clinicians. Conditions such as dementia, HF, and COPD are common and debilitating, rarely occur in exclusion, and often diagnosed late in the course of illness [86,53]. Optimal chronic disease management needs to be multifactorial and delivered by a diverse team of health care professionals [46]. To date, despite elaborate study designs, primary care interventions for elderly persons with multiple chronic conditions have demonstrated limited success [87,88,89]. Potential reasons may include targeting of complex interventions at patients too healthy to benefit, insufficient redesign of primary care processes, under-utilization of interprofessional resources, and, in some cases, limited integration of specialist resources with the patient’s existing primary healthcare system [88,89,90,91,92]. A review of ideal chronic disease management in primary care suggests that patients with chronic conditions should be stratified according to risk of poor outcomes and that the intensity of management be tailored according to patient needs [47]. With growing recognition that frailty may identify those who are at highest risk of adverse health status change [13], screening and identifying older adults who are frail allows for targeting high-intensity interventions for those who may most benefit from timely interventions directed at the co-morbid complex chronic conditions that contribute to, or are worsened by, frailty. Doing so at a primary care level may facilitate more judicious use of geriatric specialist resources, which are in short supply in Canada [2].

Adoption of this type of screening program requires a willingness within the practice setting to move from an emphasis on disease-specific programs post-diagnosis to pro-actively identifying older persons at risk, case-finding for associated conditions in those at highest risk, and early targeting of interventions for identified conditions. The advantage to pro-active screening is to identify people before substantial suffering and debility have occurred, when they may be less amenable to intervention, and to reduce risk of destabilization. Pro-active health care requires a transformation in how care of older adults is conceptualized and delivered. Typically, care for older adults is focused ‘downstream’, being reactive to destabilized health conditions, decline, or health or social crises with resulting use of acute care resources and long-term care institutionalization. In contrast, screening for frailty offers potential for an ‘upstream’ approach, aiming to reduce vulnerability by managing underlying medical conditions early, before crises occur, preventing destabilization of these conditions. Proactive optimal management of complex chronic conditions has the potential to reduce use of acute care resources, delay institutionalization and maintain quality of life for as long as possible [93,94].

### 4.1. Screening Implementation Strengths

A key strength of the C5-75 care model is that it has been feasible to implement within busy primary care practices. Level 1 implementation is coincident with already scheduled appointments with family physicians and takes less than 7 min to complete [44]. Level 2 screening requires less than 30 min and although it requires an additional appointment, the potential benefits for patients associated with identifying and managing frailty and reducing adverse outcomes through pro-active interventions may be important enough to justify the time and resource investments required for this screening. C5-75 screening at both Level 1 and Level 2 is within the nursing scope of practice and that of allied health professionals, as relevant. Dynamometer use requires minimal staff training and the cost is relatively affordable for most medical practices ($300–$400 CAD).

The C5-75 program is rooted in primary care. Family physicians are ideally positioned to assess and manage frailty and multiple chronic conditions with their ability to provide integrated care over time, particularly within interprofessional practice settings where the expertise of occupational therapists, pharmacists, and dietitians can support assessment and potentially mitigate impacts of chronic complex conditions among the elderly [33]. Collaborative interprofessional teams are considered efficient and effective in providing high-quality healthcare [95,96] and are aptly suited for care of older adults with complex chronic conditions. In this project, our ability to implement the C5-75 program was facilitated by: (a) clinic staff’s willingness to collaborate within the team; (b) a shared interest in the care of elderly adults; and (c) respect for the unique and complementary contributions that each discipline brings to the diagnostic and care planning process. Moreover, our collaborative use of a multi-disciplinary project team to develop, evaluate, and improve the program contributed to its success. Organizational support is critical for effective interprofessional collaborations [97,98].

### 4.2. Screening Implementation Limitations

Level 1 screening is implemented as part of regularly scheduled primary care visits. In some cases, patients are visiting their physicians because they are acutely ill so that screening at that time is inappropriate. Opportunities for screening are also missed if patients do not visit the office regularly; some patients who are housebound may represent the frailest of patients. This program is dependent on nursing and allied healthcare professional involvement, making it more challenging, but not impossible, to implement in solo practice settings or in settings with limited human resources. A recent study demonstrated the feasibility and acceptability of the C5-75 program as implemented in a family practice setting lacking interprofessional healthcare providers; co-located with a community pharmacy, trained pharmacy staff completed Level 1 screening and when indicated, further assessment and Level 2 intervention was completed at the family practice [99]. This study suggests potential for broader implementation of C5-75 frailty screening in primary care through collaboration with community pharmacies. 

Identifying the exact prevalence of some conditions is not always possible as not all patients who screen positive go on to completed further assessment. For example, due to physician judgement, previous testing, and patient illnesses, not all patients who screened positive for COPD completed spirometry. Moreover, it is possible that cut-offs for diagnosing some conditions may result in under- or over-diagnoses, such as those used in diagnosing COPD [100]. Currently, the extent to which recommendations are implemented by family physicians is not known; this will be examined in future studies.

## 5. Conclusions

The C5-75, Case-finding for Complex Chronic Conditions in Seniors 75+, screening program is a systematic program implemented at the primary care level to better identify frailty and other complex conditions that are challenging to identify early and, with health destabilization, often result in high health system resource use. The program re-conceptualizes the approach in primary care from reactive interventions post-diagnosis for single disease states to a proactive approach aimed at improving health outcomes, reducing suffering and maintaining best quality of life for as long as possible in the community, with the potential for reducing acute health care service utilization. Rooted in primary care practice and incorporating systematic, multidisciplinary, evidence-informed, person-centred interventions, the C5-75 program provides a significant opportunity for building capacity for care of older adults at the primary care level. Given the aging population, limited available specialist resources, and finite health system resources, comprehensive programs such as C5-75 will become of increasing importance as the health care system evolves to meet the needs of an increasing number of older adults.

## Figures and Tables

**Figure 1:** Iterative Process Used in Developing C5-75 Screening Components: Example of Heart Failure (HF) Screening. HF = Heart Failure; COPD = Chronic Obstructive Pulmonary Disease; PND = Paroxysmal nocturnal dyspnea.

**Table 1:** Summary of the objectives of the C5-75 program.

The C5-75 programs aims to: Identify individuals 75 years of age and older who are frail and thus more vulnerable and at risk of poor outcomes, often worsening and worsened by other unrecognized conditions. Identify those individuals with complex chronic conditions that are often not detected early, resulting in health destabilization and consequently resulting in high health system resource use. Optimize management of chronic complex conditions to potentially avert health destabilization with an early and pro-active approach to care; the aim is to reduce suffering and maintain best quality of life in the community for as long as possible, and potentially reduce acute care health service utilization. Use a team-based interprofessional approach to identify and manage high-risk individuals within primary care, addressing the challenge of resource limitations and limited physician time within the structure of typical family practice. Use an optimized chronic disease management approach to stratify patients according to degree of risk and tailor interventions accordingly. Provide care based in primary care practice.

**Table 2:** Case-finding for Complex Chronic Conditions in Seniors 75+ (C5-75): Level 1 Screening.

Level 1 Screening Process				
Exercise	Frailty	Heart Failure	COPD/ Worsening of COPD	Falls
Which of the following describes you best? (i) I am physically active. I do 30 min or more of moderate intensity physical activities, 5 or more days per week. (ii) I am physically active occasionally, or during some seasons much more than others. (iii) I am not physically active beyond moving around or walking during activities of daily living. If (ii) or (iii): provide Prescription for Physical Activity with information about community exercise programs.	(i) 4 m gait speed * (ii) hand grip strength ** For new assessments: If frail (gait speed ≥ 6 s): (i) Refer for Level 2 screening (ii) Refer for medication review For repeat assessments: If frail (gait speed ≥ 6 s) and if gait speed is ≥ 2 s compared to last year: (i) Refer for Level 2 screening (ii) Refer for medication review	For all persons with known heart failure: (i) Refer for Level 2 screening (ii) Refer for medication review	Do you currently smoke cigarettes or have you ever smoked cigarettes? If “yes”, ask: (i) Do you cough regularly? (ii) Do you cough up phlegm regularly? (iii) Do even simple chores make you short of breath? (iv) Do you wheeze when you exert yourself or at night? (v) Do you get frequent colds that persist longer than those of other people you know? If “yes” to any, refer for spirometry if this has not been completed within the past year.	I’d like to know about any falls you have had, whether or not you’ve had an injury. A fall means a slip or trip in which you lost your balance and landed on the floor or ground or lower level. (i) In the past 6 months, have you had two or more falls or near-falls? (Y/N) (ii) In the past 6 months, have you had a fall with injury requiring medical attention? (Y/N) (iii) If “yes” to either question: (iv) Refer for Level 2 screening (v) Refer for medication review.

COPD = Chronic Obstructive Pulmonary Disease; MD = patient’s family physician; MRC = Medical Research Council; * Gait speed instructions: “Walk at your usual speed, as if you are walking down the street to go to the store. Walk all the way past the other end before you stop”; ** Hand grip measured twice on each side: “I would like to test your grip strength on both hands, as this can be an indicator of general strength. Squeeze as tightly as you can for 3 s”.

**Table 3:** Case-finding for Complex Chronic Conditions in Seniors 75+ (C5-75): Level 2 Screening.

Level 2 Screening Process			
**Nutrition**	**Fracture Risk**	**Urinary Incontinence**	**Depression, Anxiety, Social Isolation**
Measure height Measure weight Ask: (i) Have you lost weight in the past 6 months without trying to lose weight? (ii) Have you been eating less than usual for more than a week? If “YES” to both, send to dietitian.	If on medications for osteoporosis *: MD is reminded to review appropriateness of therapy and monitoring If not on medications for osteoporosis: (i) Order X-ray T-L spine if: rib–pelvis distance ≤ 2 fingerbreadths, or Wall-Occiput distance > 5 cm, or measured height decrease of ≥ 2 cm over 3 years, or decrease of > 6 cm from patient’s tallest recalled height (ii) If ≥ 3 years since last BMD, order a BMD Ensure ≥ 800 IU Vitamin D	Ask: Do you leak urine or wet yourself? If “yes,” ask: Does it bother you enough that you’d like to have this looked into further? If “Yes”: (i) Send urine for urinalysis and culture (ii) Provide handout on avoidance of caffeine, alcohol, and excessive drinking of fluids (iii) Order post-void residual pelvic ultrasound (iv) Refer to incontinence program.	Administer PHQ-2; If score ≥ 3, request completion of PHQ-9. If PHQ-9 score ≥ 10, MD is notified 2. Administer GAD-2; If score ≥ 3, request completion of GAD-7. If GAD-7 score ≥ 10, MD is notified 3. Administer LSNS-6; If score < 12, refer for social work assessment
**Caregiver Burden** **(if applicable)**	**Cognitive Impairment**	**Falls Risk**	**Assessment Urgency Algorithm (AUA)**
If caregiver is present, administer 4-item Zarit scale (if caregiver is not present, obtain verbal consent to contact by phone). If score ≥ 8, request caregiver to complete 22-item Zarit scale If score ≥ 17, MD is notified and social work referral recommended.	If not seen in Memory Clinic within 1 year, administer Mini-Cog If positive score, MD is notified and referral to the Memory Clinic suggested	Obtain orthostatic vitals Ensure optometrist or ophthalmologist assessment within past one year Check cane/walker height Gait quality assessment Ask: (i) Do you ever feel unsteady on your feet or that you might lose your balance? (ii) Are you worried about falling? If “yes” to either question, refer to Mobility Clinic.	Administer and record score. If 6, MD is notified and recommendation made for referral to geriatric medicine.

* Medications for osteoporosis: risedronate; alendronate; denosumab; zolendronic acid; raloxifene; teriparatide. PHQ-2/PHQ-9 = Patient Health Questionnaire (2 and 9 question versions), GAD-3/GAD-7 = General Anxiety Disorder (3 and 7 item versions), BMD = Bone mineral density, LSNS = Lubben Social Network Scale.

**Table 4:** Examples of recommended management strategies for those screening positive on C5-75 program components.

Positive Screening Results	Management Recommendations
Chronic Obstructive Pulmonary Disease	Spirometry conducted by a trained respiratory therapist and diagnosis by a family physician with special training through the Spirometry in Primary Care Program, a program which has been endorsed by the Ontario Thoracic Society and Ontario Respiratory Care Society. Medical management and monitoring consistent with Canadian Thoracic Society guidelines [58]. Smoking cessation program recommended for smokers. Patient education on self-management and exercise and a COPD action plan through our respiratory therapist, who is a Certified Respiratory Educator, and a nurse and family physician who will ensure appropriate use of bronchodilators and corticosteroids as well as updated vaccinations. Based on degree of symptoms and severity, patients will be appropriately referred when necessary to local pulmonary rehabilitation programs and to respirologists for shared care.
Cognitive impairment	Referral to the Primary Care Collaborative Memory Clinic for comprehensive assessment and management using a shared care approach [74]. This includes assessment of caregiver burden, home and driving safety, future planning, exclusion of depression, delirium and other reversible causes, elimination of medications known to adversely affect cognition, introduction of cognitive enhancing medication if appropriate, and integration of community support services. Cases identified as most complex are referred to a geriatrician.
Falls risk	Referral to Mobility Clinic [75] for further assessment and management. The Mobility Clinic conducts evidence-based interprofessional assessments to determine extent of mobility and balance dysfunction, addresses reversible or modifiable factors, and initiates appropriate investigations and management. The multi-faceted approach includes sensory evaluation (vision, hearing), neurological and musculoskeletal assessment, assessment for orthostatic hypotension, analysis of footwear and gait aids, home environment review, and reduction of medications that contribute to falls risk and osteoporosis. Recommendations may include the prescription of a gait aid, alteration to home environment for safety, and individualized exercise program to improve strength and balance [76].
Low physical activity	Prescription for exercise, which includes various exercise options and a list of exercise programs available in the community.
Fracture risk	Assessment for orthostatic hypotension [77]. Management consistent with 2010 Clinical Practice Guidelines for the Diagnosis and Management of Osteoporosis in Canada for monitoring of bone mineral density [61]. Vitamin D use and regular visual assessment of cataracts as recommended by the 2011 American Geriatrics Society/British Geriatrics Society Clinical Practice Guideline for Prevention of Falls in Older Persons [60]. Assessment for correct cane or walker height, lower limb muscle weakness through chair stand test [78]; referral to the Mobility Clinic for more detailed assessment and management as necessary.
Depression, anxiety, and social isolation	Further assessment using validated tools such as the Patient Health Questionnaire (PHQ-2 and PHQ-9) [65,79], Short Anxiety Screening Test (SAST) [80], Lubben Social Network Scale (LSNS-6) [67], and Short Michigan Alcoholism Screening Test (S-MAST-G) [81], as needed. Patients identified with potential depression or anxiety are referred to their family physician for appropriate medication management and to social work counselling. For those identified as socially isolated, referral will also be made for social work assessment and possible Integrated Geriatric Service Worker support to facilitate community linkages [82].
Urinary incontinence	Further investigation for hematuria; those with hematuria are referred to a urologist. Lifestyle interventions are recommended as well as a program of pelvic floor training exercises consistent with guidelines for urinary incontinence [62,63].
Caregiver burden	Caregivers suffering from high degree of caregiver stress will be referred to a social worker with expertise in geriatrics for counselling, integration of appropriate home care supports, and future planning.
Assessment Urgency Algorithm	A function-based screening tool for assessment of risk of adverse outcome; those identified as scoring at highest risk are recommended for referral to a geriatrician [71].

**Table 5:** Characteristics of all patients screened in the C5-75 program (N = 965). In the case of multiple screens for a given patient, the most recent data point was selected.

Characteristics	Total Sample (N = 965)
Age, years, mean ± SD	81 ± 5
Gender, female, n (%)	505 (52%)
Medical history prior to screening, n (%)	
Heart Failure	299 (31%)
CAD (MI, Angina, CABG)	206 (21%)
Hypertension	482 (50%)
Diabetes	226 (23%)
Hyperlipidemia	251 (26%)
Atrial Fibrillation	107 (11%)
MCI/ dementia	104 (11%)
Osteoporosis	226 (23%)

COPD—Chronic Obstructive Pulmonary Disease; CAD—Coronary Artery Disease; MI—Myocardial Infarction; CABG—Coronary Artery Bypass Graft; MCI—Mild Cognitive Impairment.

**Table 6:** C5-75 Patient Screening Outcomes. In the case of multiple screens for a given patient, the most recent data point was selected.

Screening Component	n (%)
**LEVEL 1**	
***Screen for Exercise (N = 945)***	
I am physically active. I do 30 min or more of moderate intensity physical activities, 5 or more days per week.	453 (48%)
I am physically active occasionally, or during some seasons much more than others.	338 (36%)
I am not physically active beyond moving around or walking during activities of daily living.	154 (16%)
***Screen for Frailty (N = 965)***	
Gait speed ≤0.8 m/s	132 (14%)
Gait speed and hand grip strength *	63 (7%)
***Screen for Falls (N = 750)***	
2+ falls in the past 6 months	36 (5%)
Any falls in the past 6 months that required medical attention	26 (4%)
**LEVEL 2**	
***Fracture Risk (N = 119)***	
Prescribed medications for osteoporosis	23 (19%)
Not prescribed medications for osteoporosis but for whom T-L spine X-rays were ordered †	27 (23%)
Patients not on medications for osteoporosis for whom Bone Mineral Density testing was ordered ‡	51 (43%)
***Mental Health Screening***	
PHQ-9—positive screen for depression (N = 50)	11 (7.4%)
GAD-7—positive screen for anxiety disorder (N = 94)	4 (2.8%)
LSNS-6—positive screen for social isolation (N = 117)	29 (20%)
Zarit Caregiver Burden—positive screen for high burden (N = 103)	15 (15%)
***Cognition Screening (N = 119)***	
Mini-Cog—positive screen	26 (22%)
***Urinary Incontinence Screening (N = 147)***	
Patients reporting symptoms of urinary incontinence	47 (39%)
***Falls Risk Screening***	
2+ falls reported in the past 6 months (N = 69)	22 (32%)
Falls requiring medical attention (N = 119)	8 (7%)
***Assessment Urgency Algorithm § (N = 68)***	
Level 1	21 (31%)
Level 2	8 (12%)
Level 3	22 (32%)
Level 4	5 (7%)
Level 5	1 (1%)
Level 6	10 (15%)

Note: Number of screens for each component vary dependent on availability of complete data and when the screening protocol was introduced into the program. Insufficient data is available to report on results of screening tools that were modified or introduced recently into the protocol. Summary statistics are based on the most recent screening session for patients with multiple annual screens; PHQ-9 = Patient Health Questionnaire (9 item scale); GAD-7 = Generalized Anxiety Disorder (7 item scale); LSNS-6 = Lubben Social Network Scale (6 item scale); * Frailty defined as score within the lowest 20%, stratified by gender. † T-L spine X-rays ordered if: (i) rib–pelvis distance ≤ 2 fingerbreadths, or, (ii) W-O wall-to-occiput distance (for kyphosis) is > 5 cm, or, (iii) measured height decrease of ≥ 2 cm over 3 years, or decrease of > 6 cm from patient’s tallest recalled height. ‡ 3+ years since last test. § Higher scores reflect greater urgency for assessment due to higher risks.
